# Myocardial extracellular volume fraction and myocardial fibrosis by cmr in patients with severe aortic stenosis

**DOI:** 10.1186/1532-429X-17-S1-P290

**Published:** 2015-02-03

**Authors:** Gabriela Liberato, Juliana Bello, Rodrigo D Melo, Antonildes N Assunção Jr, Ariane B Pacheco, Breno A Soares, Debora Y Nakamura, Lea Demarchi, Victor E Costa, Roney O Sampaio, Roberto Kalil, Carlos E Rochitte

**Affiliations:** Heart Institute, InCor, University of Sao Paulo Medical School, Sao Paulo, Brazil

## Background

Severe aortic stenosis (AS) induces diffuse interstitial myocardial fibrosis (MF). CMR late gadolinium enhancement (LGE) technique is useful to detect focal MF. The amount of MF is associated with worse long-term prognosis after aortic valve replacement surgery (AVR). Measurements of T1 relaxation times before and after gadolinium administration allow determination of myocardial extracellular volume fraction (ECV) in order to assess diffuse MF. In this study we investigated ECV and LGE prevalence and extent in severe AS, and compare them to left ventricular (LV), aortic valve (AV) function and MF by biopsy.

## Methods

We analyzed 11 patients with severe AS who underwent CMR before AVR. All patients had CMR examination on 1.5 T MRI system. Patients underwent cine-MR with SSFP sequence for ventricle function evaluation (measured by Simpson's method on the short-axis images) and LGE for MF detection (by thresholding technique), using CVi42 (Circle CVi, Calgary, CA). T1 mapping used a modified Look-Locker sequence (MOLLI) before and 15-20 minutes after gadolinium administration. ECV was calculated from pre- and post-gadolinium T1 measurements of blood and myocardium, and the hematocrit. In 4 cases, ECV was compared with MF measured on myocardial biopsy obtained at AVR, stained with Picrosirius red and expressed as collagen volume fraction (CVF). χ2, Fisher exact tests, t test and Mann-Whitney test when appropriate and for correlation Spearman test were performed using Stata 12.

## Results

**Figure 1:**
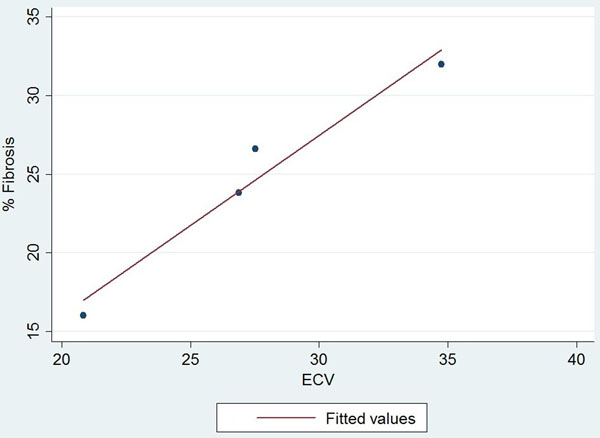
Linear correlation between ECV and quantify fibrosis from myocardial biopsy samples.

Baseline and MRI characteristics are shown on Table 1. All patients showed severe AS by aortic valve area planimetry (0.64 ± 0.27 cm2) and severe LV dysfunction (28.7 ± 11.2 %). Groups with and without LGE showed similar ECV within the remote areas (without visual LGE), 33.5% vs. 28% (p=0.12). Three patients had previous history of CAD and LGE compatible with myocardial infarction. Additionally, even when considering the entire myocardium, including infarcted areas, the ECV values remained similar between groups (30.7 vs 30.0, p=0.84). Thus, ECV measurements continued to be similar for patients with mean LGE up to 11% of LV mass. Mean myocardial fibrosis on the patients with LGE positive was 11.3% (including infarct and non-ischemic fibrosis areas). In the 4 cases with quantified myocardial biopsy, we found a very good correlation for ECV measurements between biopsy and MRI (r=0.912) (Figure 1) and the mean difference was 2.9 ± 1.8.

**Table 1:** Baseline clinical and MRI characteristics of patients with severe aortic stenosis

	ALL (n=11)	LGE positive (n=6)	LGE negative (n=5)	P - value
AGE, years +/- SD	66.2 ± 10.7	67.1 ± 12.4	65.2 ± 9.4	0.77
Male, n (%)	9 (81.8)	5 (83.3)	4 (80)	0.88
DM, n (%)	4 (36.4)	3 (50)	1 (20)	0.30
Hypertension, n (%)	10 (90.9)	5 (83.3)	5 (100)	0.33
DLP, n (%)	6 (54.5)	3 (50)	3 (60)	0.74
AFib, n (%)	3 (27.3)	1 (16.6)	2 (40)	0.38
CAD, n (%)	3 (27.8)	3 (50)	0 (0)	0.06
ACEI, n (%)	9 (81.8)	4 (66.7)	5 (100)	0.15
BBlock, n (%)	9 (81.8)	4 (66.7)	5 (100)	0.15
Labs				
Cr, ± SD	1.22 ± 0.26	1.31 ± 0.11	1.12 ± 0.09	0.24
Ht, ± SD	42.63 ± 3.61	40.6 ±2.7	44.3 ± 3.5	0.08
MRI				
LVEDVI, ml/m2 ± SD	139.8 ± 52.1	124.8 ± 52.2	157.8 ± 51.3	0.32
LVEF,% ± SD	28.7 ± 11.2	33.5 ± 8.9	23 ± 11.8	0.12
RVEDVI, ml/m2 ± SD	66.1 ± 30.9	52 ± 15.5	83.2 ± 37.8	0.06
RVEF, ml/m2 ± SD	41 ± 17.0	30.6 ± 15.0	49.6 ± 14.1	0.06
LV mass, g ± SD	212.2 ± 46.4	199.5 ± 38.1	227.4 ± 55.0	0.34
Valve area, cm2 ± SD	0.64 ± 0.27	0.68 ± 0.3	0.59 ± 0.24	0.59
Fibrosis, g± SD	11.5 ± 16.4	21.1 ± 17.1	0	0.02
Fibrosis, % ± SD	6.2 ± 9.0	11.3 ± 9.7	0	0.03
T1 native, ms ± SD	1008.6 ± 125.3	1023.3 ± 102.5	991 ± 159.3	0.69
T1 post, ms ± SD	439.9 ± 111.3	473.6 ± 54.7	399.4 ± 30.7	0.29
ECV, % ± SD	31.6 ± 5.5	32.9 ± 2.3	29.8 ± 2.4	0.38
ECV remote, % ± SD	30.2 ± 5.9	30.7 ± 4.5	30.0 ± 8.0	0.84

## Conclusions

Our initial results have demonstrated that in severe aortic stenosis, ECV measurements of the remote area were similar between patients with and without LGE. Mean ECV measurements of the entire myocardium, including LGE areas, continued to be similar between patients without LGE and with moderate LGE extent. Comparison with myocardial biopsy has demonstrated a good correlation with extracellular volume by CMR.

## Funding

FAPESP grant 2013/06149-6.

